# On the Induction of Abortion in the Vomiting of Pregnant Women

**Published:** 1852-12

**Authors:** 


*On the Induction of Abortion in the Vomiting of Pregnant Women.* By MM. Dubois and Stoltz.—During a recent discussion at the *Academie de Medicine*, M. P. Dubois stated the results of his experience in relation to obstinate vomiting in pregnancy. In proof that this is oftener a more dangerous occurrence than is usually supposed, he stated, that in the course of thirteen years he had met with twenty cases in which it has proved fatal. That obstinate vomiting is but the exaggeration of the natural sympathetic vomiting of pregnancy, and not due to any special lesion, is proved by the facts, that at the autopsies nothing is found, and that when the process of gestation becomes arrested, whether spontaneously or artifically, the vomiting is ordinarily put an end to, although the woman may not be delivered until several days after, of a dead child, and may yet die of the effects of what she has already undergone. M. Dubois refers to several cases in which the women, apparently at the point of death, were saved by the *spontaneous* death of the fœtus, this being expelled only sometime afterwards. In respect to the question of how far *artificial* interference is attended with the same result, he furnishes notes of the four cases in which he has employed it. Three of these died and one recovered—this last being added to other cases on record, making the number of recoveries he is aware of certainly 7, and probably 9. In all the cases, however, whether fortunate or not, the vomiting was suspended by the operation. The difficulty is, indeed, to fix the *period* at which this should be resorted to; for it is the natural desire to delay this as long as possible, which leads to the fatal result—the woman dying, in fact, from the exhaustion and prolonged abstinence which the vomiting has induced, prior to the operations for arresting it being undertaken. M. Dubois lays it down as a rule, never to perform it when the signs of extreme exhaustion are present, as evidenced by considerable loss of vision, cephalalgia, comatose somnolence, and disorder of the intellectual faculties. On the other hand, we should also abstain from operating when the vomiting, though violent and frequent, still allows of some aliment being retained; when the patient, though wasted and feeble, is not obliged to keep her bed; when the suffering has not yet induced intense and continuous febrile action; and when other means still remain untried. In the first case, we should not save our patient, but perhaps accelerate her death, and bring discredit on the operation; while, in the other, we should sacrifice a pregnancy that might have gone on to the full time. It is, therefore, the intermediate period that should be chosen, and this is characterized by the following signs:—1. Almost incessant vomiting, by which all alimentary substances, and sometimes the smallest drop of water, are rejected.—2. Wasting and debility, which condemn the patient to absolute rest.—3. Syncope, brought on by the least movement, or mental emotion.—4. A marked change in the features.—5. Severe and continuous febrile action.—6. An excessive and penetrating acidity of the breath.—7. The failure of all other means. But even within this period, which is of variable duration, the opportune moment must be chosen. This seems to have arrived, when the inefficacy of the most approved treatment has been proved, when fever is found to persist, and the debility and wasting of the patient are making sensible progress. The attendant should now declare that the operation is indicated, leaving to the patient and her friends the duty of deciding upon its adoption.

Professor Stoltz, of Strasburg, has published a highly interesting communication upon this subject, in which he also states his belief, that vomiting during pregnancy is much oftener fatal than is usually supposed. He relates four cases, from among others, that have come under his own notice. In three of these, death occurred, and life was saved by the operation in the fourth, although the case seemed hopeless. M. Stoltz lays great stress upon the operation being performed *in good time* because if we wait until the effects of the sympathetic reaction constitute in themselves a serious disease, the evacuation of the womb does not induce a cessation of these, and may, in certain cases, even hasten death—life, so to say, hanging upon a thread. It is undoubtedly difficult to say, *when* the moment has arrived that we can no longer hope for benefit from nature or therapeutical agents. But may not the same observation be made with regard to many important surgical operations? It is true that neither spontaneous nor artificial abortion always saves life in these cases; but the former usually occurs only when the woman's powers are hopelessly exhausted, and the pain and discharge consequent on the delivery may expedite her end—the same result not being unfrequently seen in fever. Some practitioners have expressed themselves very feelingly against sacrificing the child in these cases; but there is a great inconsistency on the part of those who do so, and who still advocate the operation in the case of narrow pelvis. A woman who has undergone artificial abortion for obstinate vomiting, may hereafter (and these cases mostly occur in primiparæ) give birth to a living child, which can never be the case in one who has so narrow a pelvis as to call for the induction of abortion rather than of premature labor.—*Bull. de l'Acad. Gazette Med.* —*Ibid.*

